# Exploring Public Perceptions of Dental Care Affordability in the United States: Mixed Method Analysis via Twitter

**DOI:** 10.2196/36315

**Published:** 2022-07-01

**Authors:** Shahen Yashpal, Ananditha Raghunath, Nihan Gencerliler, Lorel E Burns

**Affiliations:** 1 College of Dentistry New York University New York, NY United States; 2 Computer Science & Engineering University of Washington Seattle, WA United States; 3 Department of Endodontics College of Dentistry New York University New York, NY United States

**Keywords:** dentistry, oral health, social media, access to care, healthcare reform, COVID-19, dental care, health care service, twitter, public health, health communication, dental treatment, health policy, dental professional, thematic analysis

## Abstract

**Background:**

Dental care expenses are reported to present higher financial barriers than any other type of health care service in the United States. Social media platforms such as Twitter have become a source of public health communication and surveillance. Previous studies have demonstrated the usefulness of Twitter in exploring public opinion on aspects of dental care. To date, no studies have leveraged Twitter to examine public sentiments regarding dental care affordability in the United States.

**Objective:**

The aim of this study is to understand public perceptions of dental care affordability in the United States on the social media site, Twitter.

**Methods:**

Tweets posted between September 1, 2017, and September 30, 2021, were collected using the Snscrape application. Query terms were selected a priori to represent dentistry and financial aspects associated with dental treatment. Data were analyzed qualitatively using both deductive and inductive approaches. In total, 8% (440/5500) of all included tweets were coded to identify prominent themes and subthemes. The entire sample of included tweets were then independently coded into thematic categories. Quantitative data analyses included geographic distribution of tweets by state, volume analysis of tweets over time, and distribution of tweets by content theme.

**Results:**

A final sample of 5314 tweets were included in the study. Thematic analysis identified the following prominent themes: (1) general sentiments (1614 tweets, 30.4%); (2) delaying or forgoing dental care (1190 tweets, 22.4%); (3) payment strategies (1019 tweets, 19.2%); (4) insurance (767 tweets, 14.4%); and (5) policy statements (724 tweets, 13.6%). Geographic distributions of the tweets established California, Texas, Florida, and New York as the states with the most tweets. Qualitative analysis revealed barriers faced by individuals to accessing dental care, strategies taken to cope with dental pain, and public perceptions on aspects of dental care policy. The volume and thematic trends of the tweets corresponded to relevant societal events, including the COVID-19 pandemic and debates on health care policy resulting from the election of President Joseph R. Biden.

**Conclusions:**

The findings illustrate the real-time sentiment of social media users toward the cost of dental treatment and suggest shortcomings in funding that may be representative of greater systemic failures in the provision of dental care. Thus, this study provides insights for policy makers and dental professionals who strive to increase access to dental care.

## Introduction

A lack of access to dental care can lead to lower levels of systemic health, quality of life, and economic outcomes [1]. Yet, those who are most in need of dental care are often the least likely to receive it [2]. Low-income, working-age adults report the highest levels of financial barriers to needed dental care [3]. In the United States, the financial barriers to accessing dental care are higher than any other type of health care service [3-5]. The percentage of the population without dental insurance is more than twice that of those who are medically uninsured [6]. Spending on dental care results in a high percentage of out-of-pocket expenses because of a lack of insurance or high insurance deductibles and copayments [7].

While comprehensive dental coverage for children is an essential benefit under the Affordable Care Act, dental coverage for adults remains optional [7]. At the time of this study, 3 states provide no dental coverage, and 12 states provide emergency-only dental services to Medicaid beneficiaries [8]. In a comparison of public dental coverage for older adults in high-income countries, the United States had the shallowest dental coverage for older adults [9]. In an effort to mitigate access inequalities, there was a recent push in the US federal government to provide for Medicare coverage of dental and oral health services. In January of 2021, the Medicare Dental Benefit Act of 2021 (H.R.502 and S.97) was introduced into Congress, and President Biden’s budget-reconciliation package proposed funding for a Medicare dental benefit. In August 2021, the Centers for Medicare and Medicaid Services appointed a chief dental officer to guide it in advancing oral health in Medicare [10]. To best develop a policy to address financial barriers to dental care, perspectives at the individual level are needed.

Social media is increasingly becoming an essential tool for public health communication [11]. Twitter is a free social media service where people communicate their daily thoughts and behaviors in short, 280-character messages called “tweets.” With over 68 million active monthly users in the United States, Twitter offers rich, population-based data for tracking concerns of public health significance [12]. Twitter data emerge from real-world social environments, which encompass a large and diverse range of people, without any prompting from researchers. This contrasts with traditional approaches of public surveillance where responses are elicited in the form of semistructured interviews and web-based surveys with open-ended questions [13]. In addition, Twitter is a compelling data source for public health researchers because of the real-time nature of the content and high level of correlation with user sentiment and consumer confidence indices [14]. These qualities have contributed to a growing number of studies examining the use of Twitter for public health research [15-21], including the investigation of aspects of oral health [22-26]. Several studies have aimed to assess the influence of societal events on Twitter content related to oral health [27,28].

This study aims to explore the sentiments of Twitter users in the United States on dental care affordability in order to summarize trends and perceptions that describe how the cost of dental treatment impacts access to dental care. To date, no studies have leveraged Twitter to examine public sentiments regarding dental care affordability in the United States.

## Methods

### Ethical Considerations

This infodemiological study used a convergent mixed methods approach [29,30] to analyze publicly available Twitter content related to dental care affordability. This study was submitted to the Institutional Review Boards of New York University (IRB-FY2021-5634) and the University of Washington (STUDY00013725). In alignment with federal regulations regarding the use of publicly available data for research, both institutional reviews determined that the study did not meet the criteria for research involving human subjects and that no further review was required.

### Data Collection and Preprocessing

Data were obtained from Twitter, a free social media website created in 2006. Tweets can remain visible to the public or can be made visible only to approved followers, at the discretion of the user. Only publicly available tweets were used in this study, and usernames were removed for privacy protection.

Search terms were generated to identify tweets that discussed financial considerations associated with dental treatment. These search terms were tested and expanded through pilot queries and assessments until they were refined to the following word stems: “dental,” “dentist,” “tooth,” “teeth,” “root canal” AND “expensive,” “pay,” “afford,” and “money.”

Using a newly created account on Twitter, tweets were collected between September 1, 2017, and September 17, 2021, using Snscrape [31-33], an open-source web scraper written in Python (Python Software Foundation). The data collection script included a specific query that consisted of any combination of the search terms. Duplicate tweets, foreign language tweets, and retweets were then excluded. In addition to the tweets, metadata was collected as follows: “*url*,” “*date*,” “*renderedContent*,” “*user*,” “*replyCount*,” “*retweetCount*,” “*likeCount*,” “*quoteCount*,” “*retweetedTweet*,” “*quotedTweet*,” “*mentionedUsers*,” “*coordinates*,” “*place*,” “*hashtags*,” and “*cashtags*.”

Metadata related to the users’ location were used in order to limit the included tweets to those in the United States. Content was excluded from the data set of tweets for any of the following reasons: (1) content was unrelated to dental-treatment needs or experiences; (2) content was determined to be an advertisement; (3) content pertained to purely cosmetic or orthodontic dental procedures; (4) content pertained to veterinary dentistry; (5) content was classified as a joke or sarcasm; and (6) content was in reference to dental policies outside of the United States.

### Data Analysis

Data were analyzed qualitatively using both deductive and inductive approaches. Two members of the research team with clinical dental knowledge (SY and LB) co-coded all of the tweets. In total, 8% (440/5500) of all of the included tweets were coded in order to identify prominent themes and subthemes. A final codebook was developed through consensus among the members of the research team. The themes identified for the codebook included (1) general sentiments; (2) delaying or forgoing dental care; (3) payment strategies; (4) insurance; and (5) policy statements. Using the codebook, the entire sample of the included tweets was independently coded by the aforementioned researchers. In instances where the coders disagreed, they reached a consensus through discussion.

Quantitative data analysis included geographic distribution of tweets by state, volume analysis of tweets over time, and distribution of tweets by content theme. The tweets were mapped to an individual state within the United States, and the volume of tweets over time were depicted using Tableau (Tableau Software Inc), a data visualization software.

## Results

### Data Collection

**Figure 1:**
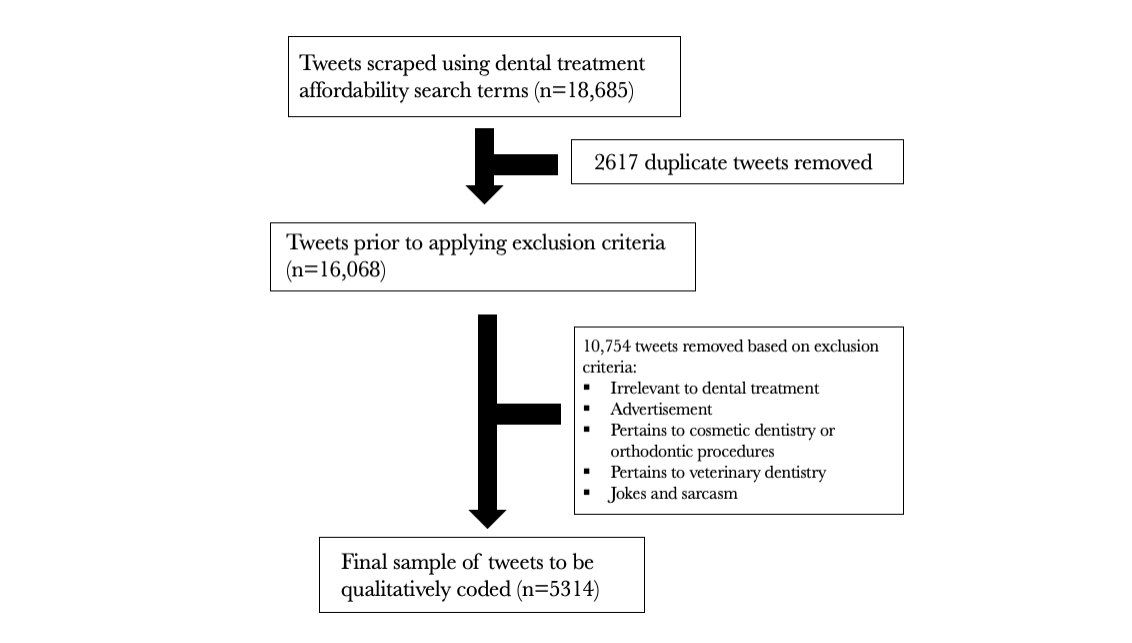
Data collection flow chart. Tweets about dental care affordability.

Using the search terms, we collected a total of 18,685 tweets from September 1, 2017, to September 30, 2021. Of these tweets, 2617 (14%) were removed as duplicates, and 10,754 (57.6%) were removed based on preestablished exclusion criteria. A final sample of 5314 (28.4%) tweets (from 4963 unique users) were included in the study (Figure 1). The top 5 pairs of search terms were as follows: dental_afford (520/5314, 9.8% tweets); teeth_pay (461/5314, 8.7% tweets); dentist_pay (436/5314, 8.2% tweets); dental_money (356/5314, 6.7%, tweets); and dental_expensive (353/5314, 6.6% tweets).

### Thematic Distribution of Tweets

Thematic analysis identified the following prominent themes (Table 1): (1) general sentiments (1614/5314 tweets, 30.4%); (2) delaying or forgoing dental care (1190/5314 tweets, 22.4%); (3) payment strategies (1019/5314 tweets, 19.2%); (4) insurance (767/5314 tweets, 14.4%); and (5) policy statements (724/5314 tweets, 13.6%). The identified subthemes and illustrative quotes from the data are presented below and in Table 2.

**Table 1:** Volume of tweets over time by thematic category.

Main category	Overall (n=5314), n (%)	2017 (n=277), n (%)	2018 (n=1227), n (%)	2019 (n=1341), n (%)	2020 (n=1090), n (%)	2021 (n=1379), n (%)
General sentiments	1614 (30.4)	110 (39.2)	399 (32.5)	405 (30.2)	320 (29.4)	380 (27.6)
Delaying or forgoing care	1190 (22.4)	64 (23.1)	304 (24.8	286 (21.3)	266 (24.4)	270 (19.6)
Payment strategies	1019 (19.2)	34 (12.3)	206 (16.8)	298 (22.2)	249 (22.8)	232 (16.8)
Insurance	767 (14.4)	44 (15.9)	173 (14.1)	187 (13.9)	155 (14.2)	208 (15.1)
Policy statements	724 (13.6)	25 (9.0)	145 (11.8)	165 (12.3)	100 (9.2)	289 (21.0)

**Table 2:** Representative tweets for theme or subtheme as described in the codebook.

Theme and subtheme		Tweet
Insurance		“My dental insurance deductible was so high, I couldn't have dental work done last year. And my knee surgery got cancelled as a result. Damn shame. Still can't afford it. [URL]” “And... 19's root canal and crown cost more than the annual dental insurance max. There go $837 of tuition money. I'm really just over this whole century.” “According to most health insurance companies teeth and eyes are luxury items that I must pay more to continue enjoying because they’re a cosmetic privilege.”
Payment strategies		“@username @username hey. I know it’s a long shot but I just had to use my rent money to have a very badly infected tooth removed and now I don’t know how I’m doing to pay rent. PLEASE help if you can 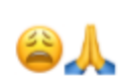 God bless ya’ll doing God’s work”^a^ “Nervous to get my tooth pulled tomorrow but mainly because I’m afraid it’s gonna cost more than what I have left on the credit card I’m using to pay for it….”
Delaying or foregoing care		“I canceled my root canal that was supposed to be Monday because my tooth quit hurting but here we are in ridiculous pain again and my husband has no job so no money to spend on it right now 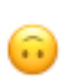 ” “@username Yes it is I have several teeth I need to get pulled/ worked on but can't afford it..and also take pain meds that don't seem to help much of anything...”^a^ “@username Do you live near a dental school? When I didn’t have dental insurance, I did an expensive project at a dental school for about 1/5 the cost. It took more time, but it was worth paying less.”^a^
**General sentiments**		
	Dentist mistrust	“I’m positive the dentist doesn’t really find cavities in my teeth they just want to get my money and torture me. How do I brush every day and floss and still get cavities every time”
	Dentistry is expensive	“@username Dental work is horribly expensive. I have had my very last $1700 root canal.”^a^
	General statements	“@username @username This is why I never want to hear another American make fun of British teeth again. People in this country cannot afford the astronomical prices charged by dentists for dental care. So they just go without dental care at all.”^a^
	Positive sentiments	“i spent a lotta money on my teeth and it was worth it 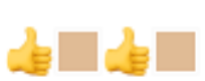 ”
Policy statements		“@DeptVetAffairs @SenateGOP @HouseGOP I cannot afford regular dental coverage as a disabled veteran with a $200 a month income.” “My mom doesn’t have insurance for dental care...now, she has an infection in her tooth... now she needs to pay $1504 before seeing the specialist... we don’t have $1504 in the bank... This is why we need Medicare For All!!! I’m angry because of this greedy corruption #Bernie2020” “@username @username When someone has a dental problem serious enough for a root canal, they are in pain and it will be an emergency treatment. But only people with money or dental insurance can get one. Most states’ Medicaid doesn’t cover adult dental care, and if it does, they pull the tooth.”^a^

^a^Username was removed to maintain the privacy of the Twitter user.

#### General Sentiments

The most prominent theme included tweets containing general sentiments that expressed that dental care was expensive or not affordable but did not additionally suggest the cost that prohibited the user from accessing needed dental care. The overwhelming majority of these tweets expressed negative sentiments. Tweets in this thematic category were subcoded into the following subthemes: mistrust of dentists, expressions that dental care is expensive for the tweeting individual or a personal acquaintance (eg, a family member), impersonal statements about the affordability of dental care, and positive sentiments about accessing dental care.

The most prevalent subtheme was “expensive dental care,” representing 70.3% (n=1135) of the tweets in this theme. Individuals frequently expressed displeasure related to the direct cost of dental care and surcharges related to administrative costs.

The consultation alone was $250... out of pocket at that ...why the dentist gotta be so expensive?? I just want to be beautiful and healthy for the low [URL].

User #677

Tweets about “dentist mistrust” represented the second most prevalent subtheme of general sentiments. Users expressed sentiments of being financially duped or perceptions of dentists prioritizing financial gain over the patient’s health.

@username @username Another common practice to make more money, is to remove all of the wisdom teeth, when often times patients do not need all of them removed. It’s just like, we’re in there, so let’s do them all.

User #1106

#### Delaying or Forgoing Dental Care

Tweets about delaying or forgoing dental care due to an inability to afford treatment were categorized into the second most prominent theme. Strategies resulting from delaying or forgoing dental care mentioned within this theme included dental tourism, visits to the emergency room, visits to free clinics or dental schools, and self-treatment.

Users tweeted about not being able to afford the upfront cost of care and as a result delaying care despite being in pain. In some cases, users shared experiences of death resulting from an inability to access dental care.

@username I had a friend who didn't get treated, he died from sepsis. I don't want to scare you but it can be infected and that can spread really fast. No dentist should refuse someone because they can't pay when it comes to infection. I wish her well.

User #1126

Users reported visiting the emergency room to address dental pain or infection when they could not afford treatment by a dentist. They often expressed dissatisfaction with their experience, which often left them with palliative care such as pain killers and antibiotics rather than treatment. In some cases, visits to the emergency room resulted in unexpected costs when medical insurances denied claims for dental-related conditions.

@username will not cover the ER visit because dental related, and I will have to pay close to $2000 hospital bill. All they did was examine me and prescribe painkiller and antibiotics. How is this right? Everything needs to change.

User #4995

Travel to countries with lower-cost dental care was expressed as a way to access care when dental treatment in the United States was considered to be too expensive. Domestically, users considered going to dental schools or public dental clinics when they could not afford the cost of a private dentist.

The fact that I’m driving three hours into Mexico tomorrow to get my teeth done at an 1/8 of the price I was going to pay at a “nonprofit” dentist in America is fucking ridiculous.

User #1990

#### Payment Strategies

The theme about “payment strategies” for needed dental care most frequently included tweets asking for donations from others. Certain twitter accounts were frequently referenced in donation requests including @pulte, @TeamPulte, and @JefferyStar. Twitter users often asked for funds to be sent to them via PayPal, Venmo, GoFundMe, and CashApp.

I'm raising money to have my teeth pulled. Click to Donate: [URL] via @gofundme.

User #241

A limited number of tweets indicated having to make sacrifices, such as foregoing groceries or rent, to afford dental care. Other payment strategies included going into debt and using stimulus checks or tax refunds to pay for care.

Really hoping I get stimulus money. I have teeth that hurt and dentists are expensive.

User #1091

#### Insurance

Tweets discussing experiences with private-payer dental insurance were included in this less prominent theme. Many tweets discussed dental insurance as an employee benefit, and some expressed gratitude for occupation-granted insurance facilitating access to dental care.

However, tweets about insurance for dental care expressed negative sentiments more frequently, including a fear of losing coverage or not being able to afford care despite having insurance. In certain instances, individuals expressed displeasure with the complexity of the insurance system and dissatisfaction for having to pay for a portion of dental care in addition to their monthly insurance premium. Some users also expressed displeasure with dental care being excluded from medical insurance.

@username I have fancy employee dental insurance, but still have to pay a bunch for crowns and root canals with the added bonus of being really limited in dentists who accept the insurance. Instead of paying $1,000 for a crown I pay $300. So basically paying $30/month for a discount card.

User #4934

#### Policy Statements

Tweets categorized as “policy statements” were the least prominent theme. The included tweets were those that mentioned Medicaid or Medicare, health care reform, were directed at politicians, or were about veterans. The content of these tweets was often related to the desired policy changes related to dental coverage.

### Time and Geographic Distribution of Tweets

Overall, 5314 tweets about dental affordability were collected (Table 1), with 277 (5.2%) in 2017, 1227 (23.1%) in 2018, 1341 (25.2%) in 2019, 1090 (20%) in 2020, and 1379 (26%) in 2021. The period of data collection was less than a year in 2017 (September 1 to December 31; 4 months) and 2021 (January 1 to September 30; 9 months).

**Figure 2:**
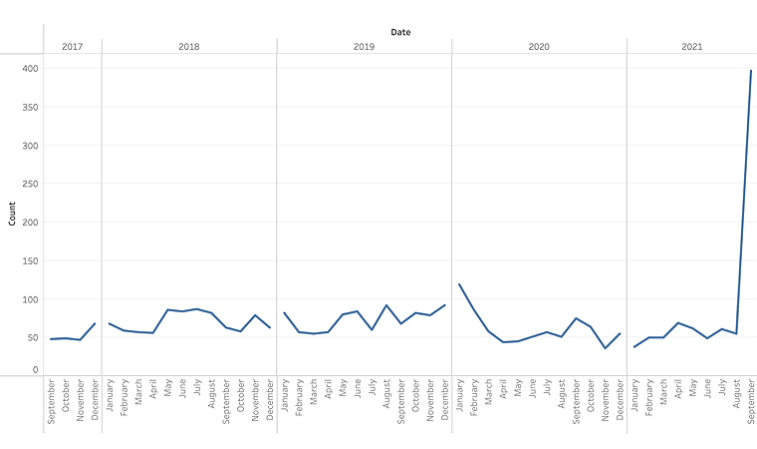
Volume of tweets by month and year.

The volume of tweets over the study period is depicted in Figure 2. There was a monthly average of 116 included tweets. The lowest number of tweets was observed in April 2020 (58 tweets, 1.1%), and the highest number of tweets was observed in September 2021 (673 tweets, 12.7%). Overall and across each year of collection, “general sentiments” was the most prevalent thematic category (Table 1). “Delaying or foregoing care” was the second most prevalent thematic category, except in 2019 and 2021, when “payment strategies” and “policy statements,” respectively, were the more frequently observed categories. “Policy statements” was consistently the least frequently observed thematic category, except for a significant increase in 2021.

**Figure 3:**
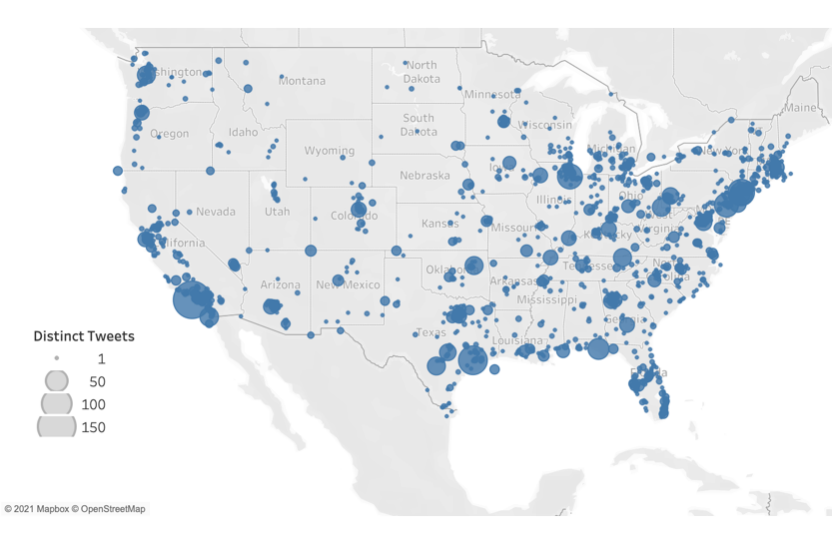
Map of tweet geolocations. Size of the dot represents the volume of tweets at a given latitude-longitude pair.

The geographic location of tweets was determined and mapped in Figure 3. Tweets originated the most from the most populous states in the United States, which are California, Texas, Florida, and New York [34].

## Discussion

### Principal Results and Comparisons With Prior Work

This study leveraged Twitter to examine public sentiments toward dental care affordability in the United States. Twitter users expressed dissatisfaction with the cost of dental treatment and the ability to access dental care. The overwhelmingly negative sentiments found in this study provide insight into how individuals are coping with financial barriers, including delaying or foregoing care and pursuing various payment strategies. Our study findings support the conclusions of the existing dental literature on dental care affordability [2-5,7]. In the 2019 National Health Interview Survey, it was found that 19.2% of women and 15.6% of men did not access their needed dental care because of cost in the prior 12 months [35]. Rates of forgoing dental care are particularly high for uninsured adults, where 1 in 2 had not seen a dentist because of costs [36].

Moreover, our study findings highlight the fact that financial barriers to accessing dental care are prevalent even among those with dental insurance. The expressed sentiments of discontent with dental insurance payment structures echoed those of the existing reports in the literature. In a study on US health care spending, among 154 conditions examined, oral disorders requiring dental care had the highest out-of-pocket costs [37]. Another study that supports the sentiments of insured tweeters in this study reported that among individuals who were insured all year, US adults were significantly more likely than adults in other developed countries to go without care because of costs, the possibility of facing high out-of-pocket spending, or the financial burden of medical bills [36].

In an effort to come up with the money required to be paid out of pocket, users reported a variety of payment strategies that included credit cards, loans, stimulus checks, tax refunds, and donations. Crowdfunding, the web-based solicitation of public donations, has become a major financer of health care–related costs [38-41]. This trend was reflected in our study findings and reinforces sentiments that the use of crowdfunding to cover direct health care expenses may be a sign of a failing system [42]. Future research may explore crowdfunding for dental procedures.

Two relevant societal events occurred during the study period, which were the COVID-19 pandemic and the election of President Biden. COVID-19 resulted in dental office closures across the country in the spring and summer of 2020, with the greatest decline in weekly visits compared with 2019 observed in the week of April 12, 2020 [43]. This trend corresponds with the quantitative findings of this study, in which the lowest number of tweets were observed in April 2020. Further, COVID-19 had an economic impact that appeared to exacerbate financial barriers to accessing dental care for some people. One user tweeted, “…I can't wait to get an eye exam and new glasses, not to mention long overdue dental care! Or just not because I can't afford it … because of #COVID19”. For others, economic stimulus checks distributed during the COVID-19 pandemic helped reduce financial barriers to dental care; for example, “@politico Got my stimulus money today. Thinking of getting much-needed dental work. Several teeth fell out when I had COVID in Dec. and Jan. Thank you President Joe!” Lastly, once dental offices reopened after COVID-19–related closures, there were tweets expressing displeasure with increased costs of care due to safety measures; for example, “…Had to pay an up charge for PPE for a dental appointment over the summer. Was told insurance was not likely to cover that aspect of the cleaning....” The election of President Biden in 2021 renewed conversations about health care reform, and the volume of tweets coded as “policy statements” increased in correspondence. In September 2021, there were 3.4 times the monthly average number of tweets. We hypothesize that this increase is a reaction to the announcement in August 2021 that President Joe Biden’s budget-reconciliation package included funding for a standard Medicare dental benefit [44-46]. Ultimately, the Medicare dental benefit was not included in the House of Representatives’ passed legislation.

### Limitations

The trends in the volume of tweets over time as well as the geographic distribution of the tweets support the generalizability and transferability of the findings of this study. However, this study is not without limitations. While all demographic categories have been shown to engage with social media to varying extents in the United States, Twitter users are not necessarily representative of the US population [47]. On average, Twitter users are younger, are more likely to identify as Democrats, are more highly educated, and have higher incomes than US adults overall [48]. Further, the sample of tweets collected may have some sampling bias, as the data set was not necessarily limited to a single tweet per unique user.

### Conclusions

The findings illustrate the real-time sentiment of Twitter users toward the cost of dental treatment and suggest shortcomings in funding, which may be representative of a greater systemic failure in the provision of dental care. Thus, this study provides insights for policy makers and dental professionals who strive to increase access to dental care. Limitations of dental insurance payment models, both public and private, are one such area that may be explored.
